# Disruption of the LTD dialogue between the cerebellum and the cortex in Angelman syndrome model: a timing hypothesis

**DOI:** 10.3389/fnsys.2014.00221

**Published:** 2014-11-19

**Authors:** Guy Cheron, Javier Márquez-Ruiz, Tatsuya Kishino, Bernard Dan

**Affiliations:** ^1^Laboratory of Electrophysiology, Université de MonsMons, Belgium; ^2^Laboratory of Neurophysiology and Movement Biomechanics, ULB Neuroscience Institut, Université Libre de BruxellesBrussels, Belgium; ^3^División de Neurociencias, Universidad Pablo de OlavideSevilla, Spain; ^4^Division of Functional Genomics, Center for Frontier Life Sciences, Nagasaki UniversityNagasaki, Japan; ^5^Department of Neurology, Hôpital Universitaire des Enfants Reine Fabiola, Université Libre de BruxellesBrussels, Belgium

**Keywords:** plasticity, cerebellum, somatosensory cortex, LTD, LTP, STDP, Purkinje cells

## Abstract

Angelman syndrome (AS) is a genetic neurodevelopmental disorder in which cerebellar functioning impairment has been documented despite the absence of gross structural abnormalities. Characteristically, a spontaneous 160 Hz oscillation emerges in the Purkinje cells network of the *Ube3a*^m−/p+^ Angelman mouse model. This abnormal oscillation is induced by enhanced Purkinje cell rhythmicity and hypersynchrony along the parallel fiber beam. We present a pathophysiological hypothesis for the neurophysiology underlying major aspects of the clinical phenotype of AS, including cognitive, language and motor deficits, involving long-range connection between the cerebellar and the cortical networks. This hypothesis states that the alteration of the cerebellar rhythmic activity impinges cerebellar long-term depression (LTD) plasticity, which in turn alters the LTD plasticity in the cerebral cortex. This hypothesis was based on preliminary experiments using electrical stimulation of the whiskers pad performed in alert mice showing that after a 8 Hz LTD-inducing protocol, the cerebellar LTD accompanied by a delayed response in the wild type (WT) mice is missing in *Ube3a*^m−/p+^ mice and that the LTD induced in the barrel cortex following the same peripheral stimulation in wild mice is reversed into a LTP in the *Ube3a*^m−/p+^ mice. The control exerted by the cerebellum on the excitation vs. inhibition balance in the cerebral cortex and possible role played by the timing plasticity of the Purkinje cell LTD on the spike–timing dependent plasticity (STDP) of the pyramidal neurons are discussed in the context of the present hypothesis.

## Introduction

### Angelman syndrome

Angelman syndrome (AS) is a genetic neurodevelopmental condition characterized by intellectual and learning disability, motor dysfunction including ataxia, speech impairment, epilepsy and typical behavioral manifestations including exuberance and easily provoked laughter. It is caused by lack of expression of the maternally inherited *UBE3A* gene, an imprinted gene located on chromosome 15Q10–Q12 (Kishino et al., 1997). Cerebellar dysfunction was suggested since the original clinical description of the syndrome (Angelman, 1965). It has been confirmed by motor studies (Dan et al., 2001; Dan and Cheron, 2004) and functional imaging (Holopainen et al., 2001; Peters et al., 2011; Tiwari et al., 2012). Mouse models with knockout maternal *Ube3a* (*Ube3a*^m−/p+^) show no morphologic cerebellar abnormalities (Jiang et al., 1998; Miura et al., 2002). However, lack of *Ube3a* expression was specifically demonstrated in the Purkinje cell layer (Miura et al., 2002; Daily et al., 2012). These mice showed ataxia (Jiang et al., 1998; Miura et al., 2002; Heck et al., 2008; Mulherkar and Jana, 2010; Jana, 2012). We previously reported the emergence of fast (160 Hz) oscillation in the cerebellum of the *Ube3a*^m−/p+^ mice (Dan et al., 2004; Cheron et al., 2005). This local field potential oscillation (LFPO) is maximum at the level the Purkinje cell layer. It is related to an increase in Purkinje cell simple spike firing and rhythmicity. The 160 Hz LFPO is highly synchronized along the parallel fiber beam. It is inhibited by gabazine and carbenoxolone (Cheron et al., 2005). In sum, it appears to be physiologically similar to that described in calcium-binding proteins knockout mice, including calretinin, calbindin and parvalbumin (Cheron et al., 2004; Servais et al., 2005). However, immunocytochemical staining demonstrated normal calbindin expression in the Purkinje cell of *Ube3a*^m−/p+^ mice (Jiang et al., 1998), this might suggest other factors than calcium signaling in the emergence of the LFPO. More recently, Egawa et al. (2012) showed that tonic inhibition is specifically decreased in cerebellar granule cells in slice recordings obtained in *Ube3a*^m−/p+^ mice. This was due related to Ube3a implication in the degradation of GABA(A) transporter 1 (GAT1) with lack of Ube3a expression inducing excess GAT1 in *Ube3a^m−/p+^* mice resulting in a decrease of GABA(A) concentration in the extrasynaptic space (Egawa et al., 2012). This default in tonic inhibition of the granule cells may increase their excitatory input onto the Purkinje cell, which is a recognized mechanism in the emergence of the 160 Hz LFPO (Bearzatto et al., 2006). In addition, a dysfunction in tonic GABA(A) conductance may explain a number of phenotypic features encountered in neurodevelopmental disorders like AS (Egawa and Fukuda, 2013).

### Neuronal plasticity

Given the cardinal learning impairments and cerebellar dysfunction in AS, the role of cerebellar plasticity has attracted increasing attention. The specific relationship between cerebellar cortex and inferior olive has recently been suggested to provide an informative avenue for integrating the evidences of neuronal plasticity in a translational perspective (Cheron et al., 2013). The Purkinje cells plastic properties are specifically controlled by input from climbing fibers, allowing processing through both feed-forward and feedback loops inside the cerebellar cortex. In addition, bidirectional connections with the basal ganglia and multiple cerebral cortex areas extend the cerebellar dynamic function toward a wide range of behaviors. More specifically, reversible inactivation of the somatosensory cortex in the rat results in a lengthening of the latency of the climbing fiber in response to a peripheral stimulus (Brown and Bower, 2002). Interestingly, it was recently demonstrated (Najafi et al., 2014) in awake mice that the climbing fiber-triggered calcium signals are enhanced when it was elicited by a sensory event allowing a strong modulation of cerebellar plasticity. The majority of brain operations necessitate the cerebellum assistance to provide exact timing of multiple signals coming from the sensory systems (Bower, 1997) and their integration in the cerebral cortex. This multi-dimensional computation would also require a timing plasticity allowing motor sequence ordering, detection of error and sensory prediction (D’Angelo and De Zeeuw, 2009; De Zeeuw et al., 2011; Heiney et al., 2014). Very recently, it was demonstrated that the L7-PP2B mice presenting impaired PC intrinsic plasticity were severely impaired in learning of an object localization task requiring a precise timing (Rahmati et al., 2014). This is of particular interest considering that the ability to produce adequate responses to sensory stimuli was preserved in this mutant. Based on these findings, these authors suggested an important role of cerebellum-cerebrum interaction in cognitive task necessitating a strict temporal tuning.

As *Ube3a* expression is present in all brain regions in principal neurons as well as GABAergic interneurons there is a great probability that a number of neuronal processes such as neuronal plasticity are compromised in AS (Gustin et al., 2010). Deficit in learning and plasticity in *Ube3a*^m−/p+^ mice have been mainly reported in hippocampal slice. Recently, following contextual fear conditioning *Ube3a*^m−/p+^ mice, Filonova et al. (2014) demonstrated a deficit in the activity-dependent increases in ERK1/2 phosphorylation, which corroborates previously reported alteration in synaptic plasticity and cognitive function in AS mice (Jiang et al., 1998; van Woerden et al., 2007; Huang et al., 2013). In particular, an abnormal LTP was demonstrated in hippocampal slice of AS mice (van Woerden et al., 2007), where following the conditioning stimuli a short time long-term depression (LTD) was recorded in place of an LTP. Moreover, these authors demonstrated that this abnormal plasticity can be rescued by introducing an additional mutation at the inhibitory phosphorylation site of alpha CaMKII. To our knowledge there is no available data about LTP or LTD in the cerebellum of the *Ube3a^m−/p+^* mice.

## Results

### Hypothesis

**Figure 1:**
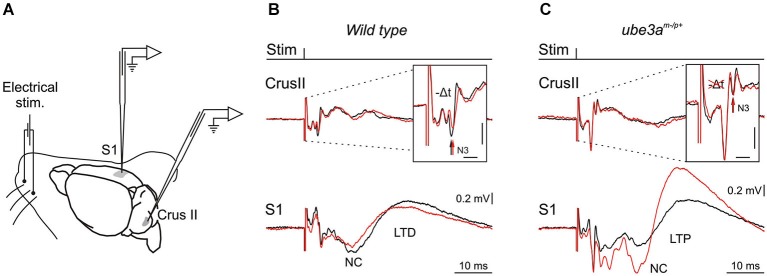
**Experimental design and electrophysiological responses to electrical stimulation of mouse whiskers during 8 Hz inducing-LTD. (A)** Animals were prepared for chronic recordings of local field potentials (LFP) and unitary extracellular activity in the Purkinje cell layer of the Crus I/II area and in S1 cortical area. Facial dermatomes of the whisker region were electrically stimulated with a pair of needles under the skin (Stim). **(B)** In the upper part, LFP recorded in the Crus II in response of a single electrical pulse before (black trace) and after 10 min of the 8 Hz LTD-inducing protocol in wild type (WT) mouse (red trace). Note the delayed and reduced N3 component (vertical arrows) after the 8 Hz LTD-inducing protocol. In the lower part, LFP recorded in the somatosensory cortex (S1) before (black trace) and after the same 8 Hz LTD-inducing protocol (red trace). Note the reduced negative component (NC) indexing a cortical LTD in WT mouse. **(C)** The same type of LFP components as in B but recorded in a *Ube3a*^m−/p+^ mouse. Note the absence of cerebellar LTD indexed by the absence of amplitude and timing change in N3 component after the 8 Hz LTD-inducing protocol (red trace) in upper part, and the reversal of the NC LTD into a NC LTP recorded in S1 (lower part). Horizontal and vertical scales in **(B)** and **(C)** correspond to 2 ms and 0.2 mV, respectively.

Here we propose a pathophysiological hypothesis stating that the cerebellar abnormal rhythmic activity recorded as fast LFPO in AS mouse model impairs cerebellar LTD plasticity, which in turn alters the LTD plasticity in the cerebral cortex. This would provide a new understanding for a range of phenotypic abnormalities seen in AS and have implications for targeted management of patients with this condition. Long range bidirectional communications as those reported between the cerebellum, the basal ganglia and the cortex highlight the importance of the precise timing operating at the different loci. Interestingly, the LTD reported in cerebellar slice (Roggeri et al., 2008) and in our recent experiments in the cerebellum of alert mice (Márquez and Cheron, 2012) were accompanied by a short delay (Figure 1B). The cerebellar LTD may influence all the chain of neuronal targets from the deep cerebellar nucleus (DCN) to the cerebral cortex via the thalamus. In order to study such possible cooperation between the cerebellar and cortical plasticity a same sensory input and a same inductor of plasticity must be used. This sensory input is in our case represented by an electrical pulse given on the whisker pad and the inductor of plasticity consists in an 8 Hz electrical stimulus given during 10 min on the whisker pad (Figure 1A). As previously reported by Han et al. (2014) such lower frequency rate (5 Hz in this case) induced also an LTD in the barrel cortex of the anesthetized rat. It seems a priori that the LTD in the barrel cortex was not accompanied by a timing plasticity as the one present in the cerebellum. How the cerebellar LTD may or not contribute to the LTD in the barrel cortex is the central question of the present hypothesis.

### Cerebellar and cerebral LTD

**Figure 2:**
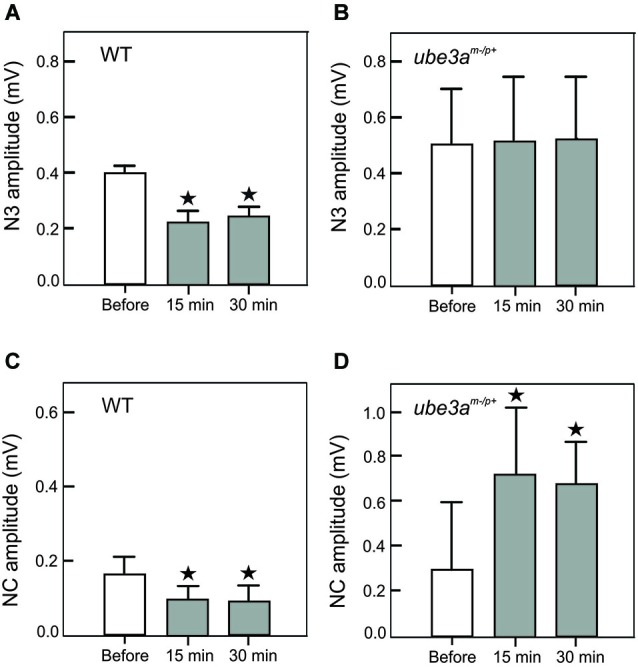
**Amplitude histogram of cerebellar N3 LFP component (A, B) and the negative component (NC) of the S1 cortical area (C, D)**. These components were recorded before, 15 and 30 min after the 8 Hz LTD-inducing protocol in WT **(A, C)** and in *Ube3a*^m−/p+^ mice **(B, D)**. Note the presence of a LTD of N3 and NC in WT mice **(A, C)** and the absence of modification of N3 **(B)** and the presence of a LTP in *Ube3a*^m−/p+^ mice. The asterisks correspond to *p* < 0.05.

This hypothesis stems from a series of results obtained using a novel LTD-inducing protocol in which 8 Hz electrical stimulation is applied on the whisker pad in the alert mouse during 10 min (Márquez-Ruiz and Cheron, 2012). A single electrical stimulus given on the whisker pad activates the afferent fibers of the trigeminal ganglion and reaches different modules of the cerebellum by the mossy fibers originating from the trigeminal nucleus (Tn) in the brainstem. From this nucleus the sensory input also reaches the inferior olive activating the climbing fiber input to the cerebellum. We have recently shown that the basic prerequisite for producing the classical LTD at the parallel fiber-Purkinje cell synapse (conjunctive stimulation of the mossy and climbing fibers; Ito and Kano, 1982; Ito, 2001) is encountered in awake mouse when single electrical stimulation was applied on the whisker pad (Márquez-Ruiz and Cheron, 2012). Indeed, this stimulation produced a first simple spike response (excitation-inhibition) shortly followed by a complex spike response (Bosman et al., 2010). This peripheral stimulus evoked a series of components in the local field of the cerebellar cortex (Márquez-Ruiz and Cheron, 2012; Figure 1B, *upper part*). Among these, the N3 component is directly related to the postsynaptic activity of the parallel fiber-Purkinje cell synapse. During the 8 Hz LTD-inducing stimulus both the simple and complex spike firing were significantly increased. Ten minutes after the 8 Hz stimulation, the amplitude of N3 peak is decreased and its latency delayed during at least 30 min (Figure 1B, *upper part*). These effects have been reproduced in the littermate wild type (WT) of the *Ube3a*^m−/p+^ mice (Figure 2A). The N3 amplitude was significantly decreased (from 0.39 ± 0.02 mV before the 8 Hz stimulation to 0.22 ± 0.04 (*p* < 0.01) 15 min after and 0.24 ± 0.03 (*p* < 0.01) 30 min after (*p* < 0.05; *n* = 4; Figure 2A). A cerebellar LTD is thus recorded in alert WT mouse. During the same time, from the Tn via the ventro-posterior medial (VPM) nucleus of the thalamus, the same electrical stimulation reaches the primary somatosensory area of the cerebral cortex (Bosman et al., 2011), where it induces a number of evoked components (Figure 1B, *lower part*). Among these, a large NC peaks around 15 ms. This NC represents compound postsynaptic activity of the pyramidal neurons. The lower part of the Figure 1B illustrates that the 8 Hz LTD-inducing stimulus specifically reduced the amplitude of the NC (from 0.16 ± 0.04 to 0.09 ± 0.03 (*p* < 0.03) 15 min after and 0.09 ± 0.04 (*p* < 0.03) 30 min after; Figure 2B). This effect thus corresponds to specific LTD of the NC evoked response. A similar evoked NC component occurring at the latency of 13–15 ms has been recently demonstrated by Han et al. (2014) in the barrel cortex of the anesthetized rat by using air puff stimulation of the whisker. These authors also demonstrated a long-lasting increase (LTP up to 60 min) of this NC after a 100 Hz conditioning stimulus and a long-lasting decrease (LTD) after a 5 Hz conditioning stimulus. This latter evidence corroborates the present cortical LTD recorded in the alert mice after 8 Hz electrical stimulus. In addition, microinjection of APV in the barrel cortex demonstrated that both LTP and LTD of the NC component are NMDA dependent (Han et al., 2014).

In *Ube3a*^m−/p+^ mice (AS mouse model), our preliminary results showed that the Purkinje cell LTD (in particular the N3 component) was absent (Dan et al., 2010), both the latency (Figure 1C, upper part) and the amplitude (Figure 2C) remained the same before and after the 8 Hz stimulation period (*p* = 0.99) In addition, we show here that a LTP of the NC was obtained in the cerebral cortex of *Ube3a*^m−/p+^ mice instead of the physiological LTD (Figure 1C, *lower part*). The NC amplitude increased from 0.30 ± 0.11 mV to 0.72 ± 0.32 (*p* < 0.05) and 0.67 ± 0.18 (*p* < 0.05) 15 and 30 min after the 8 Hz stimulation period, respectively (Figure 2D). In accordance with our hypothesis, the absence of Purkinje cell LTD is accompanied by a LTD to LTP reversal in the barrel cortex following the same peripheral stimulation and 8 Hz LTD-inducing stimulus as applied in WT controls (Figure 1B).

### Excitation vs. inhibition balance and spike–timing dependent plasticity

**Figure 3:**
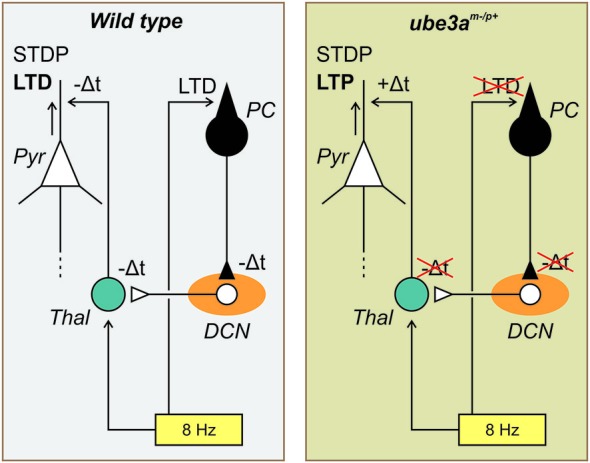
**Schematic drawing of the possible role played by the timing plasticity of the Purkinje cell LTD on the spike–timing dependent plasticity (STDP) of the pyramidal neurons**. In the WT mouse (left side), the 8 Hz LTD-inducing protocol (yellow rectangle) produce both a cerebellar LTD (at the parallel fiber-Purkinje cell (*PC*) synapse, black symbol) and a cortical LTD (in the Pyramidal cell (*Pyr*), white symbol). The PC-LTD signals is transmitted to the deep cerebellar nucleus (*DCN*, orange elipse) and via the thalamus (*Thal*, blue circle) to the somatosensory cortex. Following the present hypothesis the delayed signals (−Δ*t*) accompanying the cerebellar LTD is transmitted to the cerebral cortex where thanks to the SDTP mechanism a LTD is favored. In the *Ube3a*^m−/p+^ mouse (right side) the same diagram is represented but in this case because the cerebellar LTD is missing the delayed signals is not transmitted to the cerebral cortex resulting in +Δt inducing by the SDTP mechanism a LTP (see text for more details).

In order to account for these findings, two main mechanisms could be implicated. One concerns the control exerted by the cerebellum on the excitation vs. inhibition balance in the cerebral cortex; the other the influence of the timing of the cerebellar input on to the cerebral cortex. In terms of the excitation-inhibition balance mechanism, we hypothesize the action of the cerebellum in the control of the balance between excitatory and inhibitory neural activities within the cerebral cortex of the *Ube3a*^m−/p+^ mice may be disrupted. On the other hand, the timing plasticity of the Purkinje cell LTD may influence the spike–timing dependent plasticity (STDP) of the pyramidal neurons (Figure 3). These two possible mechanisms are not mutually exclusive but could cooperate allowing the distant action of the Purkinje cell LTD on the plasticity of the cerebral cortex.

## Discussion

### Anatomical pathways supportive of the hypothesis

The present hypothesis focused on early evoked responses both in the cerebellar Crus I-II and in the somatosensory cortex (S1). Indeed, electrical stimulation applied on the whisker pad as used in the present paradigm mainly concerned the afferent inputs conveyed by myelinated sensory fibers whose cell bodies are located either in the trigeminal ganglion or in the mesencephalic nucleus (see Bosman et al., 2011, for a review). From there the sensory input reaches the somatosensory cortex via the thalamus, and the sensory volley reaches the cerebellum by a direct path (i.e., mossy fibers) and an indirect path via the inferior olive (Cook and Wiesendanger, 1976). After integration in the cerebellar cortex and subsequently in the cerebellar module (Cerminara and Apps, 2011; Ruigrok, 2011; Llinás, 2014) the sensory message is sent back to the thalamus via the deep cerebellar nuclei. The cerebellar inputs pass through the ventral lateral (VL) complex (Asanuma et al., 1983; Aumann et al., 1994, 1996; Teune et al., 2000; Ruigrok and Teune, 2014) and the central medial nucleus of the intralaminar complex of the thalamus (Allen and Tsukara, 1974; Molinari et al., 2002) before reaching the cortex. In the rat the caudal region of the central medial nucleus projects more specifically to the sensorimotor cortex (Vertes et al., 2012).

The trigeminal input conveyed by the lemniscal and paralemniscal pathways arrives in the somatosensory (barrel) cortex mainly via the VPM and posterior medial (POM) thalamus, respectively (Viaene et al., 2011). The thalamocortical neurons of the VPM organized in “barreloids” (Lefort et al., 2009; Aronoff et al., 2010) provide about 90% of excitatory connections within layer IV and layers II/III of the cortex and convey sensory input with short latency (Killackey and Ebner, 1973; Lübke et al., 2000; Cruikshank et al., 2009; Bosman et al., 2011), In contrast, the thalamocortical POM neurons are not organized in “barreloids”. They project to layers I and V and convey sensory input with longer latency. As the present hypothesis concerns plasticity exerted on short latency responses, only the VPM contribution may be considered here.

Recent studies (Kuramoto et al., 2009) demonstrated segregation of basal ganglia and cerebellum inputs in the rat motor thalamic nuclei. The ventral anterior (VA) region mainly receives basal ganglia afferents while the VL region receives the cerebellar afferent. In addition, these regions contain two different types of thalamocortical neurons, the IZ and EZ neurons, respectively. The IZ neurons are under the inhibitory control of the internal segment of the globus pallidus and the substantia nigra pars reticulata and selectively project on the superficial cortical layer (I), whereas the EZ neurons are under the excitatory control of the deep cerebellar nuclei and selectively project to cortical layers II-V. Although, these thalamic inputs mainly concern the motor cortex (M1) they also project to the somatosensory cortex (S1; see, neurons EZ in Figure 8 of Kuramoto et al., 2009).

In addition, S1 is reciprocally connected to M1 (Ferezou et al., 2007; Mao et al., 2011). This cortico-cortical coupling between sensory and motor signals might mediate active tactile sensory perception (Ferezou et al., 2006, 2007), sensorimotor integration and motor learning (Mao et al., 2011). In addition, electrical stimulations of M1 and S1 evoked autofluorescence responses with similar time courses and amplitudes in the cerebellar cortex (Crus I; Proville et al., 2014), corroborating the well-defined corticopontine projections to the cerebellum (Leergaard et al., 2000; Suzuki et al., 2012). In this context, it was demonstrated (Popa et al., 2013) that inactivation of the cerebellum by the injection of GABAergic agonist muscimol in the cerebellar nuclei maintained a normal whisking behavior but disrupted the coherence of the gamma-band local field potentials between S1 and M1 cortices. This evidence is particularly important and in line with the present hypothesis because it demonstrates that the cerebellum may coordinate the rhythmic activities of the sensorimotor cortex.

The intracortical network is composed (1) in layer IV by the excitatory spiny stellates neurons (regular spiking) of layer IV, which are the primary targets for thalamic afferents surrounded by inhibitory basket and non-basket interneurons (fast spiking; Feldmeyer et al., 1999); and (2) in layer II/III by the excitatory pyramidal regular-spiking neurons, the fast-spiking basket and the low-threshold spiking non-basket inhibitory interneurons (McCormick et al., 1985; Gibson et al., 1999).

### Effects of cerebellar stimulation on the cerebral cortex

Cerebellar stimulation studies are in accordance with the first mechanism implicating the excitation vs. inhibition balance of the cerebral cortex. As it was demonstrated that the cerebellum may influence both inhibitory and excitatory neurons of the motor cortex in the cat (Noda and Yamamoto, 1984), monkey (Holdefer et al., 2000) and human (Daskalakis et al., 2004), we may reasonably expect that this possibility is also present in the somatosensory cortex. Cerebellar stimulation (electrical, Ugawa et al., 1991, 1994; or magnetic, Ugawa et al., 1995) applied on the lateral part of the cerebellum 5–7 ms before transcranial magnetic stimulation (TMS) of the motor cortex induces an inhibition of the motor-evoked potential. This inhibition may be provided through cortical inhibitory interneurons (Na et al., 1997) or by inhibition of thalamocortical neurons at the thalamic level (Ando et al., 1995). Interestingly, magnetic cerebellar stimulation decreases the short-interval intracortical inhibition and increases the intracortical facilitation (Daskalakis et al., 2004; Koch et al., 2008). When the inhibitory action of the Purkinje cell on the DCN is depressed, as during LTD of the Purkinje cell, the DCN could be able to activate the short-interval intracortical inhibition and to decrease the intracortical facilitation which may in turn facilitate the expression of a cortical LTD as reported after 5 Hz (Han et al., 2014) or 8 Hz (Márquez-Ruiz and Cheron, 2012) LTD-inducing protocol. Conversely, when the Purkinje cell LTD is missing, as in *Ube3a*^m−/p+^ mice, DCN output is expected to be depressed and not to be able to favor cortical LTD. On the contrary, it would activate the intracortical facilitation inducing a LTP (Figure 3).

### The STDP as a central mechanism

We propose that the reversal of the cerebral LTD into a LTP is a consequence of the disruption of the timing message controlled by the Purkinje cell LTD that could be crucial for the STDP of the pyramidal neurons of the cortex (Figure 3). The STDP is a central mechanism which occurs in the neocortex (Markram et al., 2011). It allows revisiting the LTP and LTD mechanisms at this level by highlighting the importance of a precise timing between the input and the output signals of the neocortex. The STDP was initially demonstrated by dual whole-cell voltage recordings from pyramidal neurons (Markram et al., 1997) and was independently supported by the theoretical work of Gerstner et al. (1996). It follows from this major discovery that the coincidence of the back propagation of the postsynaptic action potential and the excitatory postsynaptic potentials (EPSPs) are crucial for the induction of up or down regulation of the active synapses. Thus, we may summarize the STDP by the fact that synapses are reinforced if presynaptic spikes repeatedly occur before postsynaptic spikes within a few tens of milliseconds or less, whereas the opposite temporal order elicits synaptic weakening. In accordance with STDP mechanism we hypothesize that the delay imposed by the Purkinje cell LTD on the DCN disinhibition is transmitted to the barrel cortex where it facilitated a delayed control of the pyramidal synapse in favor of a cortical LTD. On contrary, the absence of this delayed timing in the *Ube3a*^m−/p+^ mice induces reversal plasticity toward a cortical LTP (Figure 3).

By linking output-input activities of a neuron by a strong timing constraint for plasticity, when considering the STDP, it is important to take into account all the interacting neuronal loops involved in a single behavioral plasticity. As reverberating activity (Lorente de Nó, 1938) inside of closed loop circuits and re-entrant paths are central for the understanding of Hebbian plasticity (Hebb, 1949), a local plasticity occurring in one part of the cortex (LTP or LTD) probably depends on all the input-output relationships of this part of the cortex and of the other local plasticity (LTD or LTP) occurring in other communicant networks, as for example the cerebellum. In this context, it was recently proposed that although Purkinje cells lack backpropagating Na(+) spikes, the relative timing between the climbing and parallel fibers could played a STDP at the level of the parallel fiber-Purkinje cell synapse controlling the LTP/LTD switch (Piochon et al., 2013).

The present hypothesis is supported by multiple lines of evidences supporting the existence of STDP in the somatosensory cortex (Feldman, 2000; Allen et al., 2003; Banerjee et al., 2014). Spike-timing dependent plasticity occurring at vertical layer IV and horizontal layer II/III inputs onto postsynaptic layer II/III neurons in the mouse barrel cortex has been studied and compared (Banerjee et al., 2014). They demonstrated that STDP is present for both vertical and horizontal inputs but that LTD induction at vertical inputs necessitates presynaptic NMDA supporting presynaptic LTD whereas LTD at the horizontal inputs is postsynaptic and require postsynaptic NMDA receptors. These data support the present hypothesis that cerebellar inputs could play a role in these STDP in the barrel cortex. In addition, a large scale model of the barrel cortex (Phoka et al., 2012) demonstrated that STDP can induce long-term synaptic modifications in the network encoding the dynamic features of the sensory inputs. This model corroborates the present hypothesis by the fact that via the synaptic weights modification the sensory experience reverberates into the spontaneous state dynamics which would logically integrate the presence or the absence of cerebellar plasticity.

### Plasticity in the thalamus

The segregation and relative independence of the lemniscal and cerebellar input at the thalamic level (Aumann et al., 1994, 1996) may simplify the further studies of the present hypothesis based on the interaction between cerebellar and cortical plasticity. However, we may consider the possibility that the 8 Hz stimulation of the whisker produces long-term synaptic modification at the thalamic level independently from cortical and cerebellar plasticity.

The fact that during whisker plucking, the receptive field modifications occur first in LII/III and only later or not at all in LIV layer, the site of arrival of the VPM projection, indicates that such type of plasticity occur primarily in the cortex and not in the VPM (Fox, 1994; Glazewski and Fox, 1996; Wallace and Fox, 1999).

However, this does not preclude that in case of subcutaneous block of peripheral trigeminal nerve fibers (with lidocaine), immediate plasticity occurs in the VPM, independently from the potent influence of the cortical feedback (Krupa et al., 1999). It has been proposed that the contribution of thalamic vs. cortical plasticity largely depends on the nature of factors at the origin of plasticity (Fox et al., 2000). When plasticity is induced by nerve block or lesions, thalamic plasticity is the norm (Krupa et al., 1999). Interestingly, when the nucleus gracilis of the dorsal column is destroyed, thalamic plasticity occurs but this plasticity is prevented if the cortex is ablated before inducing plasticity (Parker and Dostrovsky, 1999). In contrast, when whisker experiences are the inducing factor of plasticity, the cortical plasticity is not accompanied by measurable thalamic plasticity (Fox, 1994; Glazewski and Fox, 1996; Wallace and Fox, 1999). As the present hypothesis and related data concern whisker experience and not lesion, we may reasonably suppose that no major subcortical plasticity is concerned and that the cerebellar input remains generally unaltered when reaching the cortex. This would facilitate the further testing of the present hypothesis.

Whatever the exact role played by the olivo-cerebellar modules (motor learning vs. motor timing, Llinás, 2011), the phase locking of the PC complex spike on the 160 Hz LFP oscillation in knockout mice for genes encoding calcium binding proteins (Cheron et al., 2004) and in the *Ube3a*^m−/p+^ mice (Cheron et al., 2005) indicates that the inferior olive is influenced by the 160 Hz oscillation. This would greatly disturb the operation of the olivo-cerebellar modules and the related DCN outputs to the thalamus and the cerebral cortex. In addition, the increased PC synchrony along the parallel fiber beam recorded in the *Ube3a*^m−/p+^ mice may also influence the DCN neurons which are very sensitive to the degree of PC synchrony (Person and Raman, 2011, 2012). The presence of the cerebellar 160 Hz oscillation in *Ube3a*^m−/p+^ mice may thus be viewed as a strong pathological factor explaining the absence of cerebellar LTD and the reversal of the cerebral LTD into LTP. However, if the presence of 160 Hz LFP oscillations in the PC layer of the *Ube3a*^m−/p+^ may explain the impaired cerebellar LTD, we never recorded similar high frequency LFP oscillation in the S1 cortex of these mice (unpublished communication).

These lines of evidence seem to provide a robust avenue for testing cerebellar contribution to a number of manifestations of abnormal brain functions. In human patients with AS, several cardinal features have been related to abnormal cortical function/plasticity, including learning disability, cognitive disorder, behavioral abnormality, speech impairment and epilepsy. When testing the present hypothesis in the mouse model, there may be a case for specifically addressing the impact of high frequency oscillation on thalamo-cortical networks, and that of aberrant cerebellar LTD on cerebral plasticity. In this context, other neurophysiological abnormalities which are not specific to the cerebellum may also play a role. For example, excitatory/inhibitory imbalance has been demonstrated at both cellular and network levels in the visual cortex of *Ube3a^m−/p+^* mice (Wallace et al., 2012). In our experimental paradigm the conjunction of cerebellar and cortical LTD would logically influence the reverberating activity inside the closed loop in a way that is greatly dependent of the synaptic sign and the precise timing throughout the different loop stations. If the cortical output is depressed by a local LTD it may in turn depress the excitatory input of the cerebellar input and reinforces the LTD at the level of the Purkinje cell and thus increases the delayed disinhibition of the DCN which in turn facilitates an increase of the cerebellar input to the cerebral cortex. If this acting loop is directed to the inhibitory interneurons of the barrel cortex (Na et al., 1997; Daskalakis et al., 2004; Koch et al., 2008) it must logically facilitate the presence of the LTD in the pyramidal cell of the barrel cortex, where in the same time the delayed cerebellar excitatory input also reinforce the LTD plasticity.

The present hypothesis is also in line with the recent proposal (Watson et al., 2013) that the cerebellum may be involved in the selective relaying of “teaching signals” in order to coordinate survival and emotional behaviors through the selective relaying of “teaching signals” arising from higher centers associated with emotional behaviors.

Very recently, Proville et al. (2014) developed a new experimental paradigm using optogenetic mapping of the cerebello-cortical connections in anesthetized mice and their effect on the whisking movement in awake mice. This study elegantly demonstrates the bidirectional functional links between M1-S1 and the Crus I. On the one hand the electrical stimulation applied on S1 and or M1 induced excitatory response of PC and Golgi cells at latencies compatible with a disynaptic cortico-ponto-cerebellar connection. On the other hand, optogenetic activation of PC induced a clear switch-off of the DCN activity directly followed by excitatory responses in the thalamus and the cortex. Interestingly, during whisking the optogenetic stimulation of the PC (Crus I) triggered a backward shift of the whisking set point at about 25 ms and induced perturbation in touch control demonstrating the involvement of the cerebello-cortical loop in vibrissa-guided behavior. This timing of the PC related motor action is compatible with the present evoked field triggered by whisker at about 5 ms in the cerebellar cortex and 15 ms in S1 cortex. The latter experimental evidences are in accordance with the present hypothesis and pave the way for further investigation of plasticity dialogue along the cerebello-cortical loops.

## Conflict of interest statement

The authors declare that the research was conducted in the absence of any commercial or financial relationships that could be construed as a potential conflict of interest.
